# CD4^+^ T Cells Induced by Tuberculosis Subunit Vaccine H1 Can Improve the HIV-1 Env Humoral Response by Intrastructural Help

**DOI:** 10.3390/vaccines8040604

**Published:** 2020-10-13

**Authors:** Stephan Klessing, Vladimir Temchura, Pierre Tannig, Antonia Sophia Peter, Dennis Christensen, Roland Lang, Klaus Überla

**Affiliations:** 1 Institute of Clinical and Molecular Virology, Friedrich-Alexander-University Erlangen-Nürnberg, 91054 Erlangen, Germany; stephan.klessing@uk-erlangen.de (S.K.); pierre.tannig@uk-erlangen.de (P.T.); antoniasophia.peter@uk-erlangen.de (A.S.P.); klaus.ueberla@fau.de (K.Ü.); 2 Adjuvant Research, Dept. of Infectious Disease Immunology, Statens Serum Institut, 2300 Copenhagen, Denmark; den@ssi.dk; 3 Institute for Clinical Microbiology, Immunology and Hygiene, University Hospital Erlangen, Wasserturmstraße 3-5, 91054 Erlangen, Germany; Roland.Lang@uk-erlangen.de

**Keywords:** HIV-1 vaccine, intrastructural help, H1 tuberculosis vaccine, IgG isotype modulation

## Abstract

The induction of a potent and long-lasting, broadly neutralizing antibody response is one of the most promising approaches in HIV-1 vaccination. Recently, we demonstrated that Gag-specific T helper cells induced by DNA priming can enhance and modulate the HIV Env-specific B cell response upon virus-like particle (VLP) boost by intrastructural help (ISH). In order to minimize the induction of potentially harmful HIV specific T_H_ cells, we explored the possibility to harness the heterologous T_H_ cells induced by a recombinant tuberculosis subunit vaccine H1, which contains a fusion protein of Ag85B and ESAT-6 antigens in combination with the liposomal adjuvant CAF01. To provide ISH, immunodominant MHC-II restricted peptides from the H1 vaccine were genetically incorporated into the HIV 1 Gag protein and used for HIV VLP production. ISH effects on Env-specific antibody levels and B cell differentiation were analyzed in mice primed against H1 and boosted with VLPs. In contrast to non-primed mice, a significant increase of Env-specific IgG levels for up to 26 weeks after the last immunization was observed. This increase was largely caused by elevated IgG2b and IgG2c levels in mice that received H1 priming. Additionally, ISH enhanced the frequency of Env-specific long-lived plasma cells in the bone marrow. In this study, we were able to demonstrate that a heterologous prime-boost regimen consisting of the H1 tuberculosis subunit vaccine and T helper epitope modified HIV-1 VLPs resulted in enhanced HIV Env antibody and B cell responses, mediated by intrastructural help.

## 1. Introduction

Over the course of the last decades, there has been substantial worldwide effort in the development of a prophylactic HIV-1 vaccine. However, until now, most of the conducted clinical trials failed to confer protection [1]. Only the RV144 vaccine trial showed an estimated efficacy of 60.5% six months after vaccination, which rapidly declined to 31.2% during the following 2.5 years [2,3]. Today, it is generally believed that an induction of potent and long-lasting, broadly neutralizing antibody (bNab) response poses a highly promising approach in HIV vaccination. Surprisingly, it was previously observed that bNAbs against Env, which are thought to act in a Fab-dependent manner, also rely on subclass- and FcR-dependent mechanisms in different in vivo models [4,5]. However, the Ab response induced by Env-based immunogens is reported to be heavily dominated by the T_H_2-associated IgG1 subtype [6,7,8], although T_H_1-associated immune responses are considered more favorable for antiviral immunity due to their enhanced Fc receptor functionality [9,10]. In contrast to most investigations that focus on strategies how to best induce bNabs, we explore possibilities to modulate Fc-effector functions of antibodies to HIV Env.

While co-administration of Env-based immunogens and T_H_1-promoting adjuvants repeatedly failed to overcome the T_H_2 bias [11,12,13,14], we showed previously that induction of Gag-specific T helper cells can efficiently promote an Env-specific IgG2a dominated antibody response by intrastructural help (ISH) [15,16,17]. Different reports indicate, however, that vaccine-induced HIV-specific CD4^+^ T cells might facilitate the establishment of a systemic HIV infection [18,19,20,21,22]. To minimize the induction of HIV-specific CD4^+^ T cells, we recently harnessed T helper cells induced by the licensed vaccine Tetanol pur to enhance the Env-specific antibody response in mice [23]. In this study, the alum adjuvanted Tetanol, a strong promotor of T_H_2-associated responses [24], increased the levels of Env-specific IgG1 but not IgG2a in mice upon boosting with HIV virus-like particles (VLPs) containing MHC-II restricted tetanus peptides. This indicates that a vaccine known for the induction of a strong T_H_1 response might help to modulate the IgG subclass profile in a more favorable way.

Inextricably linked to the HIV epidemic, tuberculosis (TB), caused by *Mycobacterium tuberculosis*, is known as a major cause of mortality in HIV-1 infected individuals, showing highest incidence rates in countries with a high prevalence of HIV [25,26]. *M. bovis* bacille Calmette–Guerin (BCG) was developed as a live vaccine a century ago and is still used widely for immunization of neonates in countries with high incidence of TB. Several new vaccines for TB are in development and in clinical studies, including novel live vaccines (e.g., recombinant BCG VPM0002) and recombinant mycobacterial proteins encoded by viral vectors or combined with different adjuvants. Therefore, the recruitment of T cells induced by TB vaccination or latent infection might be a feasible approach for ISH in the context of HIV. The experimental TB subunit vaccine H1 (Ag85B-ESAT6 fusion protein) combines two immunodominant proteins of *M. tuberculosis*. In combination with the liposomal adjuvant CAF01 (cationic liposomes containing the adjuvant glycolipid trehalose-6,6-dibehenate [TDB], a synthetic MINCLE ligand), it was previously described to induce a high number of long-lasting multifunctional T_H_1 and T_H_17cells in mice and a durable T_H_1 response human [27,28,29]. Here, we investigate whether heterologous (non-HIV-1) T-helper cells induced by the experimental vaccine H1/CAF01 are able to provide intrastructural help for a long-lasting T_H_1-assosiated Env specific humoral response after immunization with HIV-VLPs containing immunodominant MHC-II restricted H1 peptides.

## 2. Materials and Methods

### 2.1. DNA Plasmids

For the H1 DNA immunizations, the previously described sequence of the H1-fusion protein with eight N-terminal histidine residues [30] was flanked by an additional unique *EcoRI* (N-terminal) and *XbaI* (C-terminal) restriction site and codon-optimized for murine translation. The resulting sequence was ordered from BioCat GmbH (Heidelberg, Germany). The introduced *EcoRI* and *XbaI* sites were subsequently used to insert the H1 sequence in the pVax vector system (Invitrogen, Carlsbad, CA, USA).

For the incorporation of the MHC-II restricted epitopes into the gag ORF, first a unique *NotI* and *AgeI* site were introduced between Q_127_ and V_128_ [31]. The resulting sequence was ordered (GeneArt, Thermo Fisher Scientific, Waltham, MA, USA) and inserted in the HgSyn backbone [32] by usage of *EcoNI* and *SfiI* restriction sites. The sequence coding for the MHC-II restricted peptides Ag85B_241–255_ (QDAYNAAGGHNAVFN) and ESAT-6_1–15_ (MTEQQWNFAGIEAAA), flanked and separated by cathepsin cleavage sites (PMGLP) [33], was ordered (GeneArt, Thermo Fisher Scientific, Waltham, MA, USA) and inserted by *NotI* and *AgeI* restriction and ligation.

### 2.2. Cell Culture

Human embryonic kidney cell line 293T/17 (HEK 293T/17, provided by Centre for AIDS Reagents, NIBSC, South Mimms, UK) were maintained in DMEM (Gibco, ThermoFisher Scientific, Waltham, MA, USA) supplemented with 10% FCS (Capricorn Scientific GmbH, Ebsdorfergrund, Germnay), 1% penicillin/streptomycin (Sigma Aldrich, Taufkirchen, Germany), and 2 mM L-glutamine (Gibco, ThermoFisher Scientific, Waltham, MA, USA) at 37 °C in a humidified 5% CO_2_ atmosphere.

### 2.3. VLP Purification

HEK 293T/17 cells were transfected at a confluence of 90% in 175 cm^2^ flasks (Greiner Bio One, Frickenhausen, Germany). For this, 40 µg of each expression plasmid encoding for pConBgp140_GCD and HgSyn-H1 was mixed with 1.25 µg of polyethylenimine (Polysciences, Warrington, PA, USA) per µg DNA and added to the cells in DMEM containing 2 mM L-glutamine. After 6 h, media was exchanged to DMEM containing 1.5% FCS, 1% penicillin/streptomycin and 2 mM L-glutamine. Two days after transfection, the supernatant was purified by sterile filtration (0.45 µm) and ultracentrifugation through a 35% sucrose cushion at 133.900× *g* and 4 °C for 2.5 h. VLPs were resuspended in sterile PBS and stored at −80 °C until further use.

### 2.4. VLP Quantification and Analysis

HIV-1 Env and Gag concentration of the purified VLPs was determined by ELISA. For this, VLPs were diluted 1:50, 1:250, and 1:1250 in bicarbonate buffer (pH 9.6) and a two-fold serial dilution of pConSgp140 (Polymun Scientific, Klosterneuburg, Austria) (starting at 500 ng/mL) and p24 (Aalto Bio Reagents, Dublin, Ireland) (starting at 1 µg/mL) was prepared. All samples were coated on high binding 96-well microtest plates (Sarstedt, Nümbrecht, Germany) at room temperature (RT)overnight. After washing the plates with PBS-T (0.1% Tween20), wells were blocked with 5% skimmed milk in PBS-T, followed by incubation with HIV-1 Env antibody 2G12 (Polymun Scientific, Klosterneuburg, Austria) or anti-p24 antibody (183-H12-5C; National Institutes of Health AIDS Reagent Program [34]) in 2% skimmed milk. After washing, HRP-conjugated antibodies directed against human or mouse IgG (Dianova, Hamburg, Germany) were added. Finally, plates were washed and relative light units (RLUs) were detected with the multilabel plate reader Victor X4 (Perkin Elmer, Hamburg, Germany).

Correct protein size in VLPs was analyzed by Western blot. Then, 3 µL of purified VLPs were separated by a 10% SDS polyacrylamide gel, followed by blotting on a nitrocellulose membrane (GE Healthcare, Little Chalfont, UK). After blockage with 5% skimmed milk in PSB-T, Env was detected by 2G12 followed by anti-human IgG-HRP incubation. Gag was stained with anti-p24 antibody and detected with anti-mouse IgG-HRP antibody staining. Chemiluminescence was captured by the Intas advanced fluorescence imager (Intas Science Imaging, Göttingen, Germany).

VLP size and PDI were analyzed by diluting the samples 1:50 in ultrapure H_2_O and measurement was performed with the ZetaSizer Nano S90 (Malvern Pananalytical, Kassel, Germany).

### 2.5. Mouse Housing and Ethic Statements

Five-week old female C57BL/6-NRJ mice were purchased from Janvier Labs (Le Genest-Saint-Isle, France) and housed in individually ventilated cages at the Franz-Penzoldt Center of the Friedrich-Alexander University Erlangen-Nürnberg (Erlangen, Germany). All conducted procedures were approved by the government of Lower Franconia under the license number 55.2-2532-2-205 according to German animal protection law.

### 2.6. Immunization

A stable formulation of CAF01 (DDA liposomes with TDB) was prepared by the lipid film hydration method as previously described [35]. For H1/CAF01 immunizations, mice were injected subcutaneously with 2 µg of H1 antigen emulsified in 100 µL CAF01 adjuvant (50 µL each flank). Before injection, the antigen was allowed to adsorb to the adjuvant for 60 min on ice.

DNA immunization was performed by intramuscular electroporation in both shaved hind legs. For this, 30 µg of plasmid DNA in PBS (0.5 µg/µL, 30 µL per leg) was injected in the center of a 2.5 mm 4-electrode array, immediately followed by an electric pulse of 63 V amplitude and 40 ms total duration (Ichor Medical, San Diego, CA, USA).

VLP boost was performed by intramuscular injection at a dose of 300 ng Env protein (5 ng/µL, 30 µL per hind leg) in sterile PBS (Gibco, Thermo Fisher Scientific, Waltham, MA, USA). Mice were immunized twice in a three week interval with H1/CAF01 or H1 DNA for the analysis of the T cell response one week after the last immunization. For the analysis of ISH effects, mice were either untreated or primed twice in a four week interval with H1/CAF01 or H1 DNA and boosted four weeks later twice in a four week interval with HIV-VLPs (H1-VLPs or WT-VLPs). Immunization schedules are summarized in the corresponding figures. All described procedures were performed at the indicated time points under anesthesia with continuous isoflurane (CP-Pharma, Burgdorf, Germany) inhalation.

### 2.7. Analysis of Cellular Immune Response

H1-specific T cell responses in the spleen were analyzed by peptide restimulation followed by intracellular cytokine staining (ICS) as previously described [16]. Briefly, after removal of the spleen, a single-cell suspension was prepared by homogenization in gentleMACS™ C Tubes and a gentleMACS™ dissociator (Milteny Biotec, Bergisch Gladbach, Germany) according to the manufacturers protocol. The cell suspension was run through a 70 µm cell strainer (Falcon, Thermo Fisher Scientific, Waltham, MA, USA). After removal of erythrocytes by incubation with ACK lysis buffer (150 mM NH4Cl, 10 mM KHCO3, 0.1 mM EDTA in H_2_O, pH 7.2) for 8 min at RT, splenocytes were resuspended in RPMI 1640 (Gibco, Thermo Fisher Scientific, Waltham, MA, USA) supplemented with 10% FCS (Capricorn Scientific GmbH, Ebsdorfergrund, Germnay), 1% penicillin/streptomycin (Sigma Aldrich, Taufkirchen, Germany), 10 mmol HEPES (Gibco, Thermo Fisher Scientific, Waltham, MA, USA), 2 mmol L-glutamine (Gibco, Thermo Fisher Scientific, Waltham, MA, USA), and 50 µmol β-Mercaptoethanol (PAN-Biotech, Aidenbach, Germany).

For ICS, 10^6^ splenocytes/well were seeded in a 96-well U-bottom microtiter plate (Greiner Bio-One, Frickenhausen, Germany) and stimulated with 10 μg/mL of the MHC-II–restricted peptides Ag85B_241–255_ (QDAYNAAGGHNAVFN) or ESAT-6_1–15_ (MTEQQWNFAGIEAAA) in the presence of 2 μg/mL anti-CD28 (37.51; eBioscience, Frankfurt am Main, Germany) and 3 μg/mL Brefeldin A (eBioscience, Frankfurt am Main, Germany) for 6 h at 37 °C in a humidified 5% CO_2_ atmosphere. After this, cells were surface stained with anti-mouse CD4 BV650 (RM4-5, Biolegend, San Diego, CA, USA) and Fixable Viability Dye eFluor 450 (eBioscience, Frankfurt am Main, Germany). Next, cells were fixed, using 2% paraformaldehyde (MORPHISTO GmbH, Frankfurt am Main, Germany) and permeabilized with 0.5% saponin (Sigma Aldrich, Taufkirchen, Germany) in the presence of 1.7 µg/mL anti-mouse CD16/CD32 (93; eBioscience). Afterwards, cells were stained intracellularly with TNFα PE-Cy7 (MP6-XT22), anti-mouse IL-2 APC (JES6-5H4), and anti-mouse IFNγ PE (XMG1.2, all from eBioscience) in 0.5% saponin. Samples were analyzed on a BD LSR-II flow cytometer (BD, Franklin Lakes, NJ, USA) and data were evaluated using FlowJo V10 (FlowJo, LLC, Ashland, OR, USA).

For the determination of antigen-specific IL-4 and IL-5 secretion by ELISA, 10^6^ splenocytes/well were incubated in complete RPMI in the presence of 2 µg/mL anti-CD28 (37.51; Life Technologies, Carlsbad, CA, USA) and 10 µg/mL of the respective peptides for 48 h at 37 °C in a humidified 5% CO_2_ atmosphere. Subsequently, supernatants were diluted 1:3 in ELISA and IL-4 and IL-5 concentration was determined by Ready-SET-Go ELISA (Life Technologies, Carlsbad, CA, USA) following the manufacturer’s guidelines.

### 2.8. Analysis of the Humoral Immune Response

Mice were bled by retrobulbar venous plexus puncture with a heparinized capillary (Hirschmann Laborgeräte, Eberstadt, Germany) regularly throughout the experiments at the indicated time-points. Blood samples were either centrifuged at 2400× *g* for 10 min in 1.5 mL reaction tubes (first experiment) or in a Microtainer SST Tube (Becton Dickinson, Franklin Lakes, NJ, USA) at 7000× *g* for 90 s (repetition). Serum was transferred into clean tubes and stored at −20 °C until further use. Env-specific antibody levels in the serum were determined by gp120 ELISA as previously described [36]. Briefly, 96 well high-binding microtiter plates were coated with 100 ng of ConBgp120-His in 100 µL bicarbonate buffer (pH 9.6) per well at room temperature overnight. After washing the plates with PBS-T (0.1% Tween20), wells were blocked with 5% skimmed milk in PBS-T for 60 min. Serum samples were diluted 1:400 in 2% skimmed milk in PBS-T and 100 µL of the diluted serum was added to each well and incubated for 60 min, followed by washing. Total IgG levels were detected by HRP anti-mouse IgG (Dianova, Hamburg, Germany). For IgG subtype-specific quantification, a two-fold serial dilution (starting with 400 ng/mL) of the gp120-specific antibody b12 fused to the murine heavy chains IgG1 or IgG2c in 2% skimmed milk was used. The respective antibody subtypes were detected by HRP-conjugated antibodies directed against IgG1, IgG2b, IgG2c, and IgG3 (Southern Biotech, Birmingham, AL, USA). Finally, the plates were washed, and relative light units were detected with the multilabel plate reader Victor X4 (Perkin Elmer, Hamburg, Germany).

Peptide-specific antibody responses were determined by coating 96 well high-binding microtiter plates with 1 µg of Ag85B_241–255_ and ESAT-6_1–15_ per well over night in bicarbonate buffer (pH 9.6) at room temperature. After blocking with 5% skimmed milk in PBS-T, diluted serum samples (1:100 in 2% skimmed milk) were added and incubated for 60 min. IgG levels were detected by HRP anti-mouse IgG and relative light units (RLU) were detected with the multilabel plate reader Victor X4.

In order to analyze the long-lived antigen-specific B cell populations, splenocytes and bone marrow cells were analyzed 26 weeks after the last immunization. Splenocytes were prepared as described above (2.7.). Bone marrow was collected by placing the femur in a punctured 0.5 mL tube, which was placed in a 1.5 mL tube and spinned down at 5000× *g* for 10 s. Cells were resuspended in complete RPMI 1640. Then, 10^6^ splenocytes or bone marrow cells/well were seeded in a 96-well U-bottom microtiter plate. After surface staining with anti-mouse CD45R/B220 APC (RA3-6B2, BD, Franklin Lakes, NJ, USA), anti-mouse CD138 PE (281-2, Biolegend, San Diego, CA, USA), anti-mouse CD19 PE-Cy7 (1D3, BD, Franklin Lakes, NJ, USA), and Fixable Viability Dye eFluor450 (eBioscience, Frankfurt am Main, Germany). Splenocytes were additionally surface stained with ConBgp120-His AlexaFluor488, which was generated with the Alexa Fluor™ 488 protein labeling kit (Thermo Fisher Scientific, Waltham, MA, USA). Cells were fixed in 2% PFA. Bone marrow cells were stained intracellularly with ConBgp120-His AlexaFluor488 in 0.5% Saponin. Samples were analyzed on an Attune NXT flow cytometer (Thermo Fisher Scientific, Waltham, MA, USA) (ISH Exp. 1) or on a BD LSR-II flow cytometer (BD, Franklin Lakes, NJ, USA) (Repetition) and data were analyzed using FlowJo V10 (FlowJo, LLC, Ashland, OR, USA).

### 2.9. In Vitro T Cell Activation

Splenic B cells from naïve C57BL/6 mice or PGT-121 BCR transgenic mice [37] were isolated by magnetic cell separation following the manufacturer’s instructions (Miltenyi Biotec, Bergisch Gladbach, Germany, #130-090-862). Then, 10^5^ B cells were incubated with VLPs equal to 10 ng Env (H1- and WT-VLPs) or the corresponding amount (8.47 ng) of p24 (HgSyn-H1) for 4 h at 37 °C. Single cell suspension of splenocytes of H1/CAF01 immunized mice was prepared as described above. Then, 1 × 10^6^ splenocytes were seeded in the presence of 3 μg/mL Brefeldin A (eBioscience, Frankfurt am Main, Germany). Either 10^5^ B cell/VLP mixture or 10 µg/mL Ag85B_241–255_ and ESAT-6_1–15_ peptides were added and incubated for 16 h at 37 °C in a humidified 5% CO_2_ atmosphere. Cells were prepared for intracellular cytokine staining as described under 2.7. and analyzed on a BD LSR-II flow cytometer (BD, Franklin Lakes, NJ, USA).

### 2.10. Statistical Analysis

Statistical analysis in the form of one-way ANOVA followed by Tukey’s multiple comparisons test or Dunnett’s multiple comparisons test, Kruskal–Wallis test with Dunn’s post test was performed with GraphPad Prism Software 6 (Graphpad Software Inc., San Diego, CA, USA) as indicated in the figure legends.

## 3. Results

### 3.1. Comparison of T Cell Responses Induced by H1/CAF01 or H1-DNA Immunization

**Figure 1:**
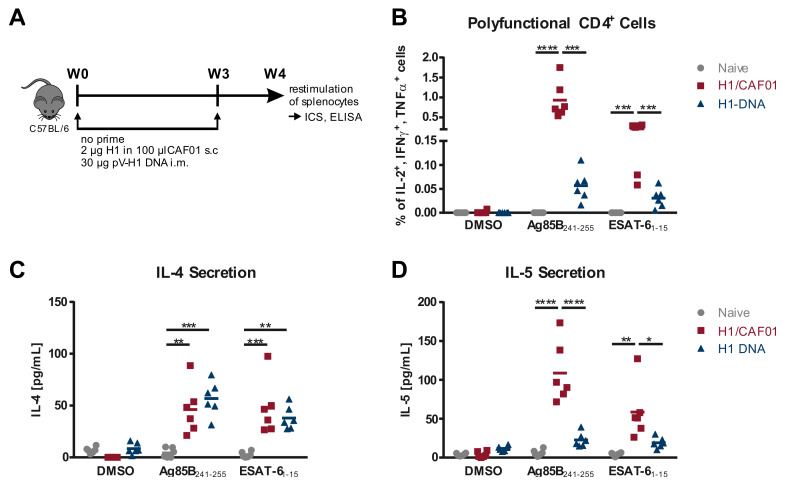
**Characterization of the T helper cell response after H1 Immunization.** (**A**) Six week-old female C57BL/6 mice were immunized twice in a three week interval with 2 µg of recombinant H1 protein in 100 µL CAF01 subcutaneously, or 30 µg of pV-H1 DNA by i.m. electroporation. One week after the last immunization, splenocytes were collected and restimulated with 10 µg/mL of the indicated peptide. (**B**) Shown are the frequencies of polyfunctional (IL-2+, IFN-γ+, TNFα+) CD4+ cells as determined by Boolean gating (Figure A1A). Secretion of IL-4 (**C**) and IL-5 (**D**) after 48 h culture of splenocytes in presence of 10 µg/mL peptide. Individual values of six mice per group (*n* = 6) with an indication of the mean values are depicted. Significance was determined for each stimulation by one-way ANOVA followed by Tukey’s multiple comparisons test (* *p* < 0.05; ** *p* < 0.01 *** *p* < 0.001; **** *p* < 0.0001).

**Figure A1:**
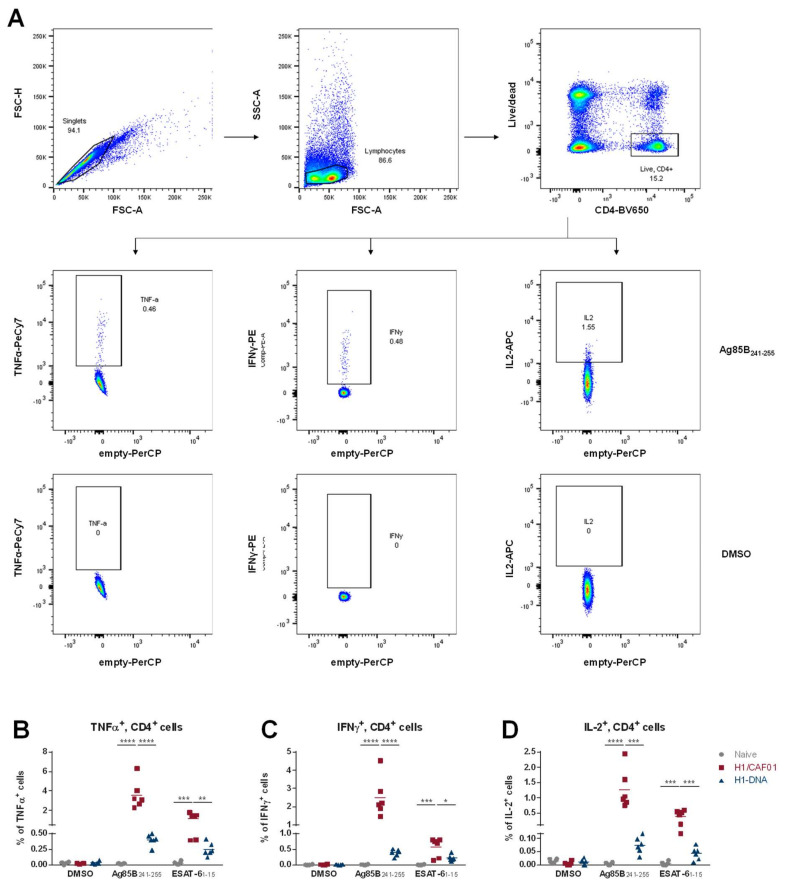
**Gating Intracellular Cytokine Staining and individual levels of cytokines**. Spenocytes were surface stained after restimulation with anti-mouse CD4 BrilliantViolet650 and Fixable Viability Dye eFluor 450. After fixation, cells were permeabilized with 0.5% saponinin the presence of 1.7 µg/mL anti-mouse CD16/CD32. Afterwards, cells were stained intracellularly with anti-mouse TNFα PE-Cy7, anti-mouse IL-2 APC), and anti-mouse IFNγ PE in 0.5% saponin. (**A**) Shown are representable dot plots from one H1/CAF01 immunized mouse, restimulated as indicated with AG85B_241–255_ or DMSO control. Boolean gating was used to identify frequencies of polyfunctional cells (TNFα^+^, IL2^+^, IFNγ^+^). Individual values for TNFα^+^ (**B**) IFNγ^+^ (**C**) and IL-2^+^ (**D**) CD4^+^ cell frequencies of six mice per group (*n* = 6) with an indication of the mean values are depicted. Significance was determined for each stimulation by one-way ANOVA followed by Tukey’s multiple comparisons test (* *p* < 0.05; ** *p* < 0.01 *** *p* < 0.001; **** *p* < 0.0001).

We previously reported on the possibility to recruit T cells induced by the licensed vaccine Tetanol pur to provide help for HIV Env-specific B cells, resulting in elevated T_H_2-associated IgG1 levels [23]. In contrast, Tetanus DNA control priming led to a modest increase of T_H_1 associated IgG2a levels, setting focus on the T cell response induced by different vaccinations in the context of ISH. Here, we now investigate whether the induction of a T_H_1-dominated primary T cell response can mediate a shift by ISH towards a more desirable IgG2c-dominated Env-specific antibody response. Therefore, we first compared the T cell response induced by the M. tuberculosis subunit vaccine candidate H1 in combination with the liposomal adjuvant CAF01 to H1 DNA immunization. For this, mice were immunized twice in a 3-week interval with H1/CAF01 or a H1 DNA expression plasmid. Antigen specific T cell responses were determined one week after the last immunization by in vitro restimulation of splenocytes with previously described immunodominant peptides Ag85B_241–255_ and ESAT-6_1–15_ [28,30] (Figure 1A), followed by intracellular cytokine staining (Figure 1B, Figure A1) or cytokine-specific ELISA (Figure 1C,D). As expected, we were able to observe a strong induction of polyfunctional T_H_1 cells in mice that were immunized with H1/CAF01, especially upon Ag85B_241–255_ restimulation. Further, also DNA immunized mice showed an elevated frequency of polyfunctional CD4+ T cells after restimulation with both candidate peptides, compared to naïve mice (Figure 1B). Both peptides seem to induce comparable levels of IL-4 secretion in all immunized mice (Figure 1C), whereas only in H1/CAF01 immunized mice a notable IL-5 secretion was observed (Figure 1D). However, compared to the observed IL-4/IL-5 levels secreted after Tetanol Pur immunization, the levels of T_H_2 cytokines detected here are strongly decreased [23]. Taken together, these results indicate that a restimulation with both candidate peptides induces a strong T_H_1 biased immune response in all immunized mice, with a stronger response in H1/CAF01 immunized mice.

### 3.2. Generation of Peptide-Bearing VLPs

**Figure 2:**
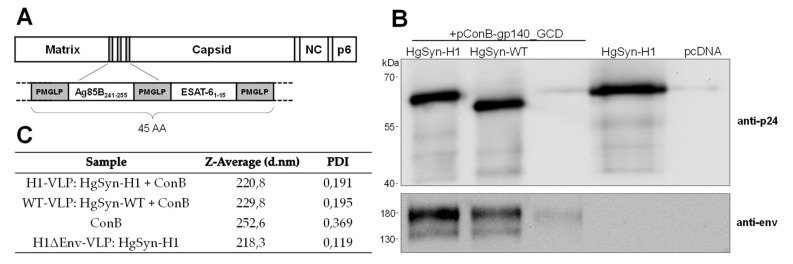
**Generation and Analysis of H1 Peptide-bearing VLPs.** (**A**) Schematic overview of the HIV Gag protein encoded by HgSyn-H1 (**B**) Representative Western blot analysis of 3 µL of purified particles after co-transfection of HgSyn-H1 or HgSyn-WT and pConB-gp140_GCD, or after single transfection of pConBgp140_GCD, HgSyn-H1 (0.5 µL), or pcDNA3. Blot was stained first with HIV-1 Env specific 2G12 antibody and anti-human IgG-HRP, followed by anti-p24 and anti-mouse IgG-HRP. (**C**) Z-average values and PDI (polydispersity index) as determined by ZetaSizer of 1:50 diluted particles in ultrapure H2O.

Next, the coding sequences for both candidate peptides were inserted in frame into the codon-optimized HIV-1 Gag expression plasmid HgSyn [31] between the Matrix and Capsid protein (Figure 2A). In order to enhance antigen presentation, both encoded peptides are flanked by the cathepsin cleavage site PMGLP [32]. Particle formation was analyzed and compared to HgSyn-WT after VLP purification by ultracentrifugation. Here, Western blot analysis (Figure 2B) showed similar levels of expression of both Gag variants when co-transfected with the HIV-1 Env expression plasmid pConB-gp140_GCD. Further, the increased size of the detected Gag band indicates a successful incorporation of the candidate peptides. The detected Env signal was also comparable between both constructs, as well as hydrodynamic size, and PDI (polydispersity index) as determined by ZetaSizer (Figure 2C). When transfected alone, ConB gp140_GCD seems to be incorporated at low levels into exosomes with a high dispersity, while HgSyn H1 alone seems to form a high number of uniform particles (Figure 2B,C). Taken together, these results show that the incorporation of the peptides did not affect the particle formation and that particles could be purified in sufficient amount to be used in immunization experiments.

### 3.3. Analysis of VLP Uptake, Processing, and Peptide Presentation

**Figure 3:**
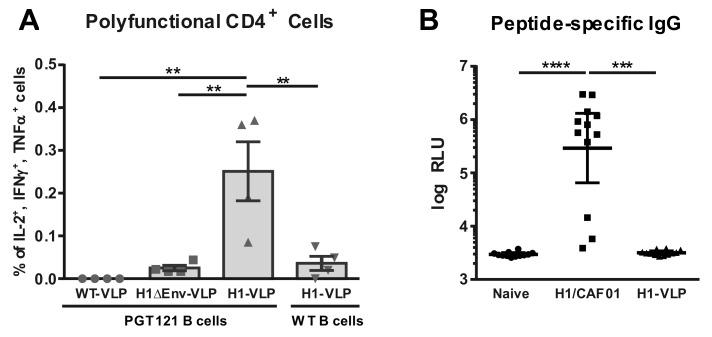
**Env-specific VLP Uptake and Presentation of Peptides.** (**A**) B cells of WT or PGT-121 BCR transgenic mice were incubated with WT-VLPs, H1-VLPs, or H1ΔEnv-VLP and subsequently cocultured with splenocytes of four H1/CAF01 immunized C57BL/6 mice. Shown are percentages of polyfunctional (IL-2^+^, IFN-γ^+^, TNFα^+^) CD4^+^ cells as determined by Boolean gating (Figure A1A) for individual mice, where the bar represents the mean values with SEM. (**B**) Peptide-specific IgG levels of 12 naïve, H1/CAF01 or H1-VLP immunized C57BL/6 mice determined by ELISA. Depicted are individual RLU values with an indication for the geometric mean with 95% CI. Statistical differences were determined by Kruskal–Wallis test with Dunn’s multiple comparisons test (* *p* < 0.05, ** *p* < 0.01, *** *p* < 0.001, **** *p* < 0.0001).

Since ISH is based on the recognition of MHC-II restricted peptides presented by Env-specific B cells, the uptake of VLPs and presentation of peptides remained to be analyzed. For this, B cells isolated from naïve WT or Env-specific PGT-121 BCR transgenic mice were incubated with WT-VLPs, H1-VLPs, and H1-VLPs without Env (H1ΔEnv-VLP). After uptake of VLPs, B cells were co-cultured with splenocytes from H1/CAF01 immunized mice. Intracellular cytokine staining and flow cytometric analysis revealed that only VLPs which contain H1 peptides internally as well as Env on their surface lead to a restimulation of T_H_1 cells (Figure 3A), while there is no restimulation after incubation with WT-VLPs and H1ΔEnv-VLP. Further, there is only a minor response observable when VLPs were incubated with naïve WT B cells.

As a subdominant immunogen, HIV Env might be susceptible to antigenic competition by other immunodominant epitopes [33,34]. By genetic incorporation of peptides into the gag ORF, H1 peptides should only be present internally in the VLPs, thereby preventing induction of peptide specific B cells. To verify the masking of H1 peptides, we analyzed peptide-specific IgG levels in mice immunized twice with either H1/CAF01 or H1-VLPs by peptide specific ELISA. Here, we observed only background IgG levels in H1-VLP immunized mice, while H1/CAF01 immunization leads to a significant increase of peptide specific IgG response (Figure 3B).

Thus, these results indicate an antigen-specific uptake of VLPs as well as correct processing and presentation of incorporated H1 peptides by Env-specific B-cells in vitro. In vivo application of H1-VLPs did not induce anti-peptide antibodies.

### 3.4. H1 Priming Immunization Enhances Env IgG Response by Intrastructural Help

**Figure 4:**
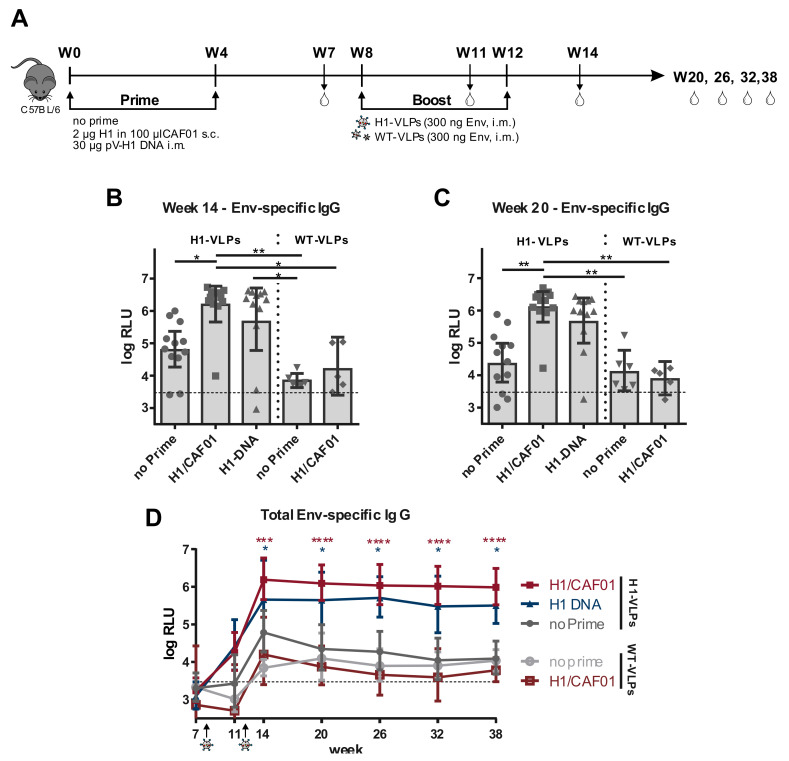
**Heterologous Prime-Boost Immunization enhances Env-specific IgG Response.** (**A**) Six-week old C57BL/6 mice were either untreated or primed twice in a 4 week interval with 2 µg H1 in 100 µL CAF01 s.c. or 30 µg pV-H1 DNA i.m. After four weeks, all mice were boosted either with H1-VLPs (HgSyn-H1/pConBgp140-GCD) or WT-VLPs (HgSyn-WT/pConBgp140-GCD) adjusted to 300 ng Env i.m. Mice were bled over the course of the experiment, as indicated by the drop symbols. (**B**–**D**) Env-specific IgG levels as determined by ELISA with 1:400 diluted serum samples and detection by anti-mouse IgG. Shown are individual log-transformed RLU values with indication for geometric mean and 95% CIafter background subtraction for 11–12 mice of two independent experiments (H1-VLP boosted) or 5–6 mice (WT-VLP boosted, 1 experiment) 2 (**B**) or 8 (**C**) weeks after the last booster immunization. Cut-off of the ELISA is indicated by the dotted line, as mean of cut-offs for all time points. Significance was determined by Kruskal–Wallis test with Dunn’s multiple comparison test. (**D**) Geometric mean of log-transformed RLU (relative light units) values for all time points with 95% CI. Significance to non-primed and H1-VLP boosted mice was determined for each time point by Kruskal–Wallis test with Dunn’s multiple comparison test (* *p* < 0.05; ** *p* < 0.01; *** *p* < 0.001; **** *p* < 0.0001).

In order to analyze whether the detected high frequency of T_H_1 cells induced by H1/CAF01 can also enhance the Env-specific antibody response upon VLP boost, mice were immunized with a heterologous prime/boost regimen. C57BL/6 mice were either untreated or primed twice in a four week interval with H1/CAF01 or H1 DNA. Four weeks after the second prime, all mice were boosted twice in a four-week interval with H1-VLPs (HgSyn-H1/pConB-gp140_GCD) or WT-VLPs (HgSyn-WT/pConB-gp140_GCD) (Figure 4A). Mice were bled routinely and Env-specific IgG serum levels were determined by ELISA. Two weeks after the last immunization, (Figure 4B) significantly higher Env-specific IgG serum levels were detectable in all primed mice that received H1-VLP boosting, compared to unprimed mice. In addition, H1/CAF01 primed mice boosted with WT-VLPs showed similarly low levels of Env-specific IgGs compared to mice, which received only WT-VLPs, indicating only a minor role of the adjuvant itself in the induction of Env-specific antibodies. Further, there is only a modest increase of Env-specific IgGs visible in mice only immunized with H1-VLPs compared to WT-VLP immunized mice, which levels out over time and is not present 8 weeks after the last immunization (Figure 4C). Monitoring Env-specific IgG levels over a longer time shows that in these mice, Env-specific IgG levels stay comparably low, whereas Env-specific IgG levels in primed mice are significantly increased up 26 weeks after the last immunization (Figure 4D). Taken together, these results indicate that Env-specific IgG levels can be enhanced by intrastructural help using H1/CAF01 and matched VLPs.

### 3.5. H1/CAF01 Immunization Can Modulate Env-Specific IgG Isotypes

**Figure 5:**
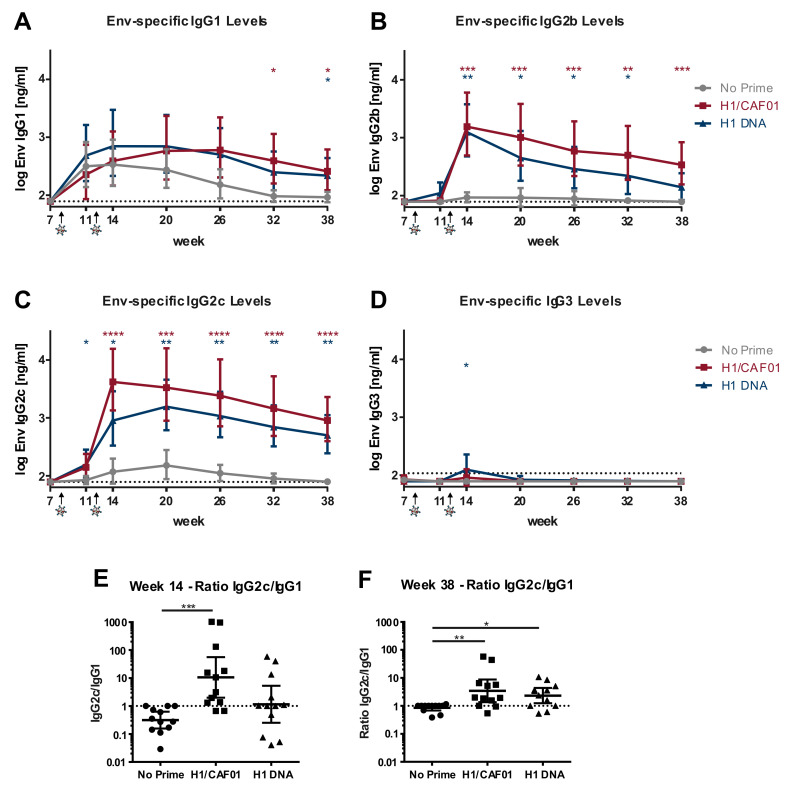
**Env-specific IgG subtypes induced by ISH.** Shown is the geometric mean [ng/mL] of log-transformed values with 95% CI of 11–12 mice of two independent experiments for (**A**) IgG1, (**B**) IgG2b, (**C**) IgG2c, (**D**) IgG3 levels. ELISA plates were coated with ConBgp120, blocked and incubated with 1:400 diluted serum samples, followed by detection with the respective subtype-specific anti-mouse IgG-HRP antibody. IgG concentration was determined by usage of the gp120-specific antibody b12 fused to the respective murine heavy chain of the different IgG subtypes in a two-fold serial dilution. Significance to non-primed mice was determined for each time point by Kruskal–Wallis test with Dunn’s multiple comparisons test (* *p* < 0.05; **p < 0.01; ***p < 0.001; **** *p* < 0.0001). (**E**,**F**) Ratio of [ng/mL] IgG2c/IgG1 values two weeks (**E**) or 26 weeks (**F**) after the last immunization, displayed as individual values with indication for geometric mean and 95% CI. Significance to non-primed mice was determined by Kruskal–Wallis test with Dunn’s multiple comparisons test (* *p* < 0.05; ** *p* < 0.01; *** *p* < 0.001; **** *p* < 0.0001).

Since H1 immunization can increase Env-specific IgG levels by intrastructural help, we were interested whether different immunization strategies can overcome the Env-induced IgG1 bias towards an IgG response dominated by isotypes known for their increased FC-receptor functionality and antiviral activity [9,10]. For this, the serum samples of H1-VLP boosted mice were analyzed by IgG subtype-specific Env ELISA. The gp120-specific antibody b12 fused to the respective murine heavy chain was used as a standard along with detection by subtype-specific anti-mouse IgG HRP antibodies. While there was no difference visible in Env-specific IgG1 levels between the groups early after the immunization, H1/CAF01 and H1 DNA primed mice seem to have slightly elevated IgG1 levels for a longer time compared to unprimed mice (Figure 5A). Moreover, a very strong increase of IgG2b and IgG2c levels in H1/CAF01 primed mice compared to non-primed mice after H1-VLP boost can be observed (Figure 5B,C) for up to 26 weeks after the last immunization. Further, also H1-DNA immunized mice showed significantly elevated IgG2b and IgG2c levels compared to non-primed mice. However, only few of H1-DNA primed mice showed a detectable Env-specific IgG3 response 2 weeks after H1-VLP boost (Figure 5D).

Visualized by the ratio of IgG2c to IgG1, a clear shift of the antibody subclasses to an IgG2c dominated antibody response in most H1/CAF01, but only some H1 DNA primed mice can be observed (Figure 5E). Background IgG levels were set to the detection limit, thereby leading to an IgG2c/IgG1 ratio of 1 at late time points in non-primed mice (Figure 3F). Taken together, these results indicate that H1/CAF01 is able to modulate the HIV-1 Env-specific immune response by intrastructural help to a more desirable IgG2b and IgG2c dominated response.

### 3.6. H1/CAF01 Priming Enhances Frequency of Long-Lived Plasma Cells

**Figure 6:**
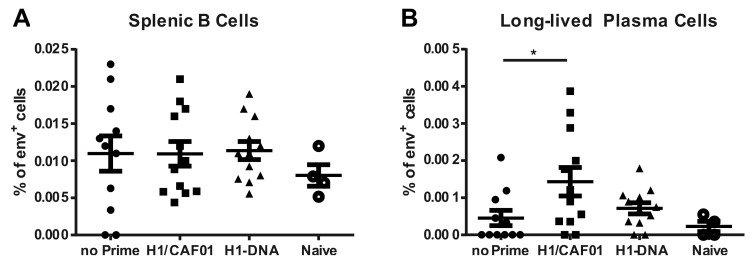
**Influence of H1 on Env-specific B cell populations.** (**A**) Frequency of Env-specific splenic B cells defined as living CD138^−^, CD19^+^, B220^−^, Env^+^ cells as determined by flow cytometric analysis (Figure A2A) 26 weeks after the last immunization. (**B**) Long-lived plasma cells isolated from the bone marrow defined as living, CD19^−^, B220^−^, CD138^+^, Env^+^ cells (Figure A2B). Shown are individual values for 4 (naïve) or 11–12 animals of two independent experiments with indication for mean and SEM. Significance to H1/CAF01 primed mice was determined by ordinary one-way ANOVA with Dunnett’s multiple comparisons test (* *p* < 0.05).

**Figure A2:**
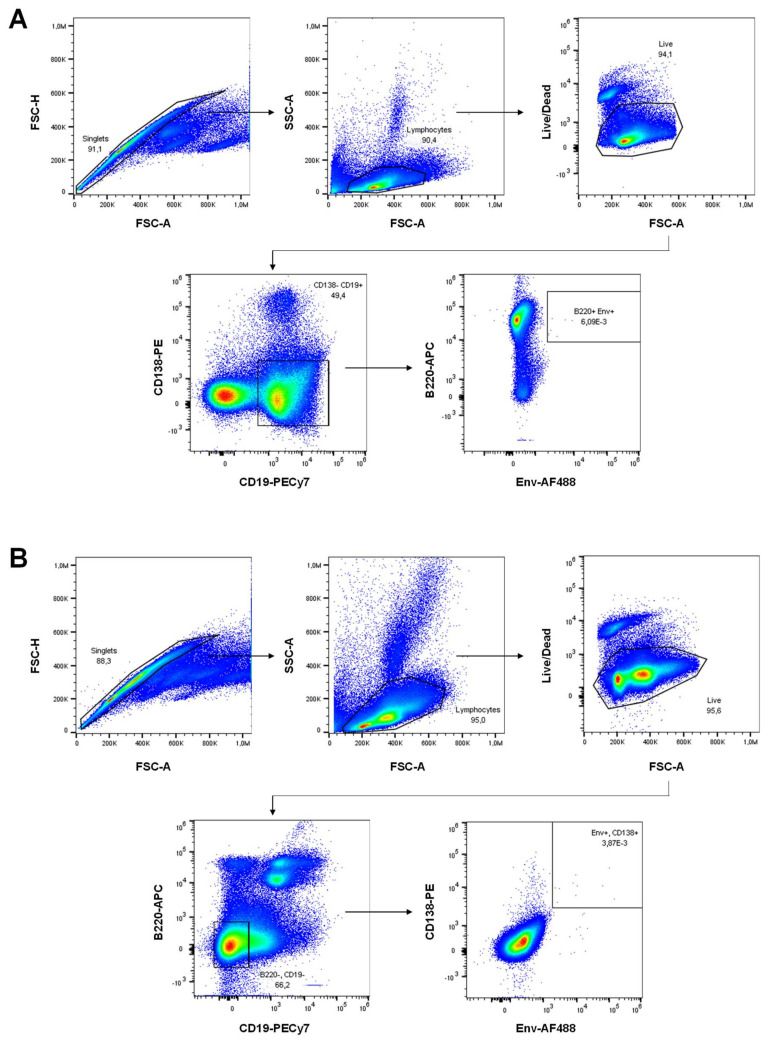
**Gating B cell staining**. (**A**) Spenocytes were surface stained with anti-mouse CD45R/B220 APC, anti-mouse CD138 PE, anti-mouse CD19 PE-Cy7, Fixable Viability Dye eFluor450 and ConBgp120-His AlexaFluor488. (**B**) Bone marrow cells were surface stained with anti-mouse CD45R/B220 APC, anti-mouse CD138 PE, anti-mouse CD19 PE-Cy7, Fixable Viability Dye eFluor450. After fixation, intracellular staining with ConBgp120-His AlexaFluor488 in 0.5% saponin was performed. Shown are dot plots from one representable H1/CAF01 primed and H1-VLP boosted mouse.

Since priming with H1/CAF01 drastically increased the serum IgG levels, we investigated next whether also the frequency of certain Env-specific B cell populations in spleen or bone marrow was affected. Therefore, splenocytes and bone marrow cells were isolated 26 weeks after the last immunization and stained against CD19, B220, CD138 and with a fluorescently labeled ConB-gp120 (Figure A2). All groups showed similar levels of Env-specific splenic B cells (Figure 6A). However, elevated frequencies of Env^+^ long-lived plasma cells in in the bone marrow can be observed in H1/CAF01 immunized mice, compared to non-primed or naïve mice (Figure 6B). These data indicate an impact of intrastructural help on the generation of long-lived plasma cells.

## 4. Discussion

As the only HIV-1 glycoprotein, the Envelope protein poses the most accessible target for a vaccine-induced antibody response. While the humoral response of most HIV-1 patients is largely dominated by the T_H_2 associated IgG1 subtype [38,39,40], a T_H_1 associated IgG3 Env-specific antibody response is correlating with virus control in elite controllers [41,42,43,44,45]. Additionally, the RV144 trial revealed IgG3 levels as correlate of protection, further emphasizing the importance of IgG subtypes and their Fc functionality in mediating protection against HIV-1 [46]. With an efficacy of 60.5% six months after vaccination that decreased to only 31.2% after 3 years [2,3], the need for novel vaccination strategies inducing a sustained high level of functional antibodies becomes evident.

VLPs offer several beneficial properties as a potential vaccine platform. Due to their spherical structure of approx. 200 nm diameter, they closely mimic infectious viral particles and can efficiently activate innate and adaptive immune responses [47,48,49]. However, HIV VLPs mostly lead to a short-lived IgG1 dominated response (Figure 5), which might be due to the induction of short-lived secondary plasma cells [50].

In an initial approach with HIV WT-VLPs, we were able to demonstrate that Gag-specific T cells can modulate the quality and strength of the Env humoral response by intrastructural help (ISH). The induction of these Gag-specific T cells might however facilitate the risk of infection [18,19,20,21,22]. To circumvent this, we previously showed that also heterologous T cells induced by the licensed vaccine Tetanol Pur can enhance the Env-specific IgG1 response in mice by intrastructural help [23].

In the present study, we now demonstrate that the T helper cell response induced by the tuberculosis subunit vaccine H1 in combination with the liposomal adjuvant CAF01 can shift the HIV-1 Env antibody response to an IgG2b and IgG2c dominated response (Figure 5). These isotypes are considered most potent in regard to Fc effector functionality and antiviral immunity in mice [51,52,53,54]. In contrast to the strong T_H_2 phenotype after Tetanol Pur immunization, we and others were able to observe a strong T_H_1 response induced by H1/CAF01 immunizations (Figure 1, [28,29]), emphasizing the role of T cell responses induced by different priming strategies in the context of ISH. Here, it is generally accepted that a T_H_1 response promotes an IgG2c isotype switch by IFNγ, which was also shown to inhibit IgG2b and IgG3 production [55]. Since we observed a strong induction of both IgG2b and IgG2c (Figure 5B,C), it is likely that also other T helper subsets, which were not analyzed in this study, contribute to the observed ISH effects. Here, especially, T_H_17 cells were shown to induce an IgG2b isotype antibody response [56]. Although not analyzed in this study, previous reports describe an induction of T_H_1 as well as T_H_17 cells upon H1/CAF01 immunization [27,30,35], offering a potential link between the observed HIV-specific IgG2b levels and H1/CAF01 immunization.

The T cell dependent induction of long-lived plasma cells is thought to take place in the germinal centers, where especially T follicular helper cells (Tfh) can provide help to B cells [57]. The reported induction of Tfh by CAF01 immunizations [58], further supports the observed increased frequency of long-lived plasma cells after H1/CAF01 prime (Figure 6B). Taken together, these results indicate that an in-depth analysis of the T cell response induced by different priming strategies might help to further determine an optimal priming strategy in order to modulate the ISH effects in the most desirable way.

In this study, H1/CAF01 as well as H1 DNA priming proved to induce desirable ISH effects, although the former seemed superior compared to H1 DNA priming. Despite the overall efficacy of DNA prime in the preclinical ISH mouse models [16,23], it might be difficult to translate it into clinical trials, due to relatively weak immunogenicity of DNA vaccines in humans [59].

Although the model of ISH presented here is based on a vaccine system currently investigated in clinical trials, a recent report by Ng et al. also indicates a possible clinical applicability of ISH with the licensed tuberculosis vaccine BCG [60]. The authors demonstrate that a fusion protein of an immunogen and two BCG peptides (one of which was similar to this study) is able to recruit BCG-induced T helper cells, leading to an isotype switch to the IgG2c subtype. In line with the results presented in this study, this indicates that BCG-induced T cells may also be harnessed to provide ISH to Env-specific B cells upon booster immunizations with VLPs, containing matched epitopes. Among currently licensed antiviral vaccines, the recombinant HBV vaccines HEPLISAV-B^®^ containing the adjuvant CpG 1018 (a proprietary toll-like receptor (TLR)- 9 agonist) as well as Fendrix^®^, comprising the adjuvant AS04™ (composed of Aluminium hydroxide and monophosphoryl lipid A, a TLR-4 agonist) also demonstrated a polarized T_H_1 response [61,62,63,64]. A direct comparison of the differently adjuvanted HBV vaccines regarding their ability to induce and modulate ISH might further broaden a clinical potential for the ISH approach.

In context of weak immunogens such as the HIV-1 Env glycoprotein, it has been previously described that antigenic competition can impair the Env antibody response induced by fusion proteins [65,66]. In contrast to fusion proteins of antigens and T_H_ epitopes, the encapsulation of immunodominant peptides into VLPs offers the benefit of preventing the induction of peptide-specific antibodies (Figure 3B), while simultaneously improving BCR crosslinking by the antigen presented on the surface of the VLPs [67,68,69]. Additionally, since ISH is based on a pre-existing immune response, masking of T_H_ epitopes might be crucial, to avoid boosting of antibody responses to these epitopes raised during the priming immunization. In addition, other enveloped nanoparticle platforms, such as liposomes, which can be easily produced under standards required for clinical applicability, pose an intriguing alternative to VLPs in order to exploit intrastructural help [70].

Taken together, we demonstrated here that, based on our previous studies on ISH, a rationally designed ISH protocol allows for the induction of a long-lasting Env antibody response with a high frequency of Env-specific long-lived plasma cells. We observed a clear shift in the Env-specific antibody isotypes to a more desirable IgG2b and IgG2c dominated response, which was mediated by heterologous (non-HIV) CD4 T cells. This opens new possibilities to further improve vaccination efficacy in the field of HIV vaccination. Since H1/CAF01 is not licensed yet, a search for alternative priming strategies among licensed vaccines composed with novel, non-alum adjuvants might improve clinical applicability.
